# Are the mechanisms driving somatosensory reorganization cortical or subcortical?

**DOI:** 10.3389/fnana.2014.00062

**Published:** 2014-07-04

**Authors:** Andrei Mayer de Oliveira, Felipe Cabral Miranda

**Affiliations:** Instituto de Biofísica Carlos Chagas Filho, Universidade Federal do Rio de JaneiroRio de Janeiro, Brasil

**Keywords:** amputees, neuronal plasticity, spinal cord lesions, somatosensory cortex, sprouting

It has been long known that somatosensory deafferentation can produce a dramatic reorganization of the somatotopic map, characterized by the retraction of the deafferented body part representation followed by expansion of unaffected body part representations (Pons et al., 1991). Mechanisms driving this phenomenon are not clear, nor is it evident whether they occur within the cortex and/or at subcortical structures (Florence et al., 1998; Jones and Pons, 1998; Jain et al., 2000). The occurrence of anatomical alterations in the cortex after deafferentation (Florence et al., 1998), in addition to the notion that neocortex is a very plastic structure, led to the view that cortical reorganization of sensory maps after lesions is driven, at least in part, by cortical mechanisms. Recent work published by Kambi et al. (2014) contradicts this paradigm. In order to determine the extent to which different sites of somatosensory pathway potentially contribute to cortical plasticity, Kambi and colleagues lesioned the dorsal column in monkeys. They then mapped the hand representation in area 3b during inactivation of the cortical face region or the cuneate nucleus. They showed that transient inactivation of normal chin representation in area 3b did not affect the expanded chin representation, even in the vicinity of the former face/hand boundary. Surprisingly, inactivation of the cuneate nucleus completely abolished responses of the expanded chin representation. These results suggest that after lesions of the dorsal column, reorganization in area 3b is dependent on plastic alterations in the brainstem, and not in the cortex. In fact, cortical reorganization was probably mediated by growth of trigeminal axons into the cuneate nucleus, as previously shown by Jain et al. (2000).

The apparent absence of corticocortical mechanisms driving cortical receptive field reorganization in these experiments is very intriguing. Simultaneous recordings from the normal chin and deafferented body representation of S1 demonstrated the expansion of the chin area in animals with dorsal column lesions (Kambi et al., 2014). Based on previous studies, it would be expected that this was due to new corticocortical connections, at least in the vicinity of the face/hand border. Moreover, large-scale sprouting of cortical connections following forelimb deafferentation has already been shown by Florence et al. (1998). This divergence in the results might be related to the type of deafferentation. In Kambi et al. (2014), animals underwent a lesion in the dorsal column, which only interrupts the ascending somatosensory information from the forelimb. Unlike amputees or individuals that suffered complete sectioning of the spinal cord, they could still move their forelimb and consequently, the motor representation of the forelimb was still present. Accordingly, Kambi et al. (2011) has shown that the forelimb motor representation in M1 is substantially preserved after lesion of the dorsal column. Perhaps this is the key difference between models in which cortical mechanisms do or do not contribute to the reorganization of cortical maps.

The hand representation in area 3b receives inputs from M1 (Liao et al., 2013). This direct feedback, as well as indirect inputs from other cortical areas, may be a potential source to keep cortical hand region activated during forelimb movements even after lesions of the dorsal column. In this scenario, maintenance of activity by area 3b cortical inputs would preclude production of signals that induce corticocortical sprouting, and so maintain the segregation between face and hand regions. This would explain why large-scale sprouting in area 3b was observed by Florence et al. (1998), but apparently not by Kambi et al. (2014). In that study, animals had amputations and at some point, they lost the motor representation of the missing body part. Accordingly, in humans that have suffered complete spinal cord injury, the reorganization of the somatosensory cortex also results from growth of new lateral connections in the cortex (Henderson et al., 2011). It is possible that depending on the type of deafferentation, the mechanisms driving the functional reorganization in the cortex can be called into action at different levels of the somatosensory system. It would be interesting to explore this question by using the same experimental protocol as Kambi et al. (2014) in amputee animals. Additionally, injections of neurotracers could be done into the deafferented cortical region in order to determine whether or not sprouting occurs after different types of deafferentation.

Dendritic spine loss has also been described in deafferented cortical neurons after spinal cord injury (Ghosh et al., 2012). Previous work has shown that alterations in dendritic spine morphology occur after lesions in the central nervous system (Keck et al., 2013) and may differ depending on the site of reorganization and type of sensory deprivation (Whitt et al., 2014). Perhaps synaptic plasticity after lesions of the dorsal column, as performed by Kambi et al. (2014), may induce different cellular responses compared to other types of deafferentation. Additionally, the lack of signal in expanded chin representation after lidocaine infusion in cuneate, but not in area 3b raises the intriguing possibility that these two sites are independently regulated and may present different molecular features in response to lesions. Studies concerning such morphological alterations and the key molecular players behind them would shed light on the location and mechanisms of plastic changes after different types of lesion.

Finally, differences in mechanism driving cortical reorganization after deafferentation may also correlate with manifestation of phantom limb pain. It has been proposed that this phenomenon is caused by a maladaptive plasticity in the somatosensory cortex (Ramachandran, 1993). Nevertheless, the occurrence of cortical sprouting, as well as nuances in synaptic plasticity after different types of deafferentation, may account for the development of phantom pain. If so, different types of deafferentation may demand different strategies for treatment. Interestingly, phantom pain is especially common in amputees. Perhaps this is due to specific cortical mechanisms (e.g., cortical sprouting) that are absent in other types of lesion (e.g., lesion of the dorsal column). A better understanding of the differences in mechanisms driving cortical reorganization between different types of deafferentation may provide valuable data for developing therapies to alleviate phantom limb pain.

## Conflict of interest statement

The authors declare that the research was conducted in the absence of any commercial or financial relationships that could be construed as a potential conflict of interest.

## References

[B1] Florence S. Taub H. Kaas J. (1998). Large-scale sprouting of cortical connections after peripheral injury in adult macaque monkeys. Science 282, 1117–1121 10.1126/science.282.5391.1117 9804549

[B2] Ghosh A. Peduzzi S. Snyder M. Schneider R. Starkey M. Schwab M. E. (2012). Heterogeneous spine loss in layer 5 cortical neurons after spinal cord injury. Cereb. Cortex 22, 1309–1317 10.1093/cercor/bhr191 21840844

[B3] Henderson L. A. Gustin S. M. Macey P. M. Wrigley P. J. Siddall P. J. (2011). Functional reorganization of the brain in humans following spinal cord injury: evidence for underlying changes in cortical anatomy. J. Neurosci. 31, 2630–2637 10.1523/JNEUROSCI.2717-10.2011 21325531 PMC6623700

[B4] Jain N. Florence S. L. Qi H. Kaas J. H. (2000). Growth of new brainstem connections in adult monkeys with massive sensory loss. Proc. Natl. Acad. Sci. U.S.A. 97, 5546–5550 10.1073/pnas.090572597 10779564 PMC25865

[B5] Jones E. G. Pons T. P. (1998). Thalamic and brainstem contributions to large-scale plasticity of primate somatosensory cortex. Science 282, 1121–1125 10.1126/science.282.5391.1121 9804550

[B6] Kambi N. Halder P. Rajan R. Arora V. Chand P. Arora M. (2014). Large-scale reorganization of the somatosensory cortex following spinal cord injuries is due to brainstem plasticity. Nat. Commun. 5, 1–10 10.1038/ncomms4602 24710038

[B7] Kambi N. Tandon S. Mohammed H. Lazar L. Jain N. (2011). Reorganization of the primary motor cortex of adult macaque monkeys after sensory loss resulting from partial spinal cord injuries. J. Neurosci. 31, 3696–3707 10.1523/JNEUROSCI.5187-10.2011 21389224 PMC3079898

[B8] Keck T. Keller G. B. Jacobsen R. I. Eysel U. T. Bonhoeffer T. Hübener M. (2013). Synaptic scaling and homeostatic plasticity in the mouse visual cortex *in vivo*. Neuron 80, 327–334 10.1016/j.neuron.2013.08.018 24139037

[B9] Liao C. C. Gharbawie O. A. Qi H. Kaas J. H. (2013). Cortical connections to single digit representations in area 3b of somatosensory cortex in squirrel monkeys and prosimian galagos. J. Comp. Neurol. 521, 3768–3790 10.1002/cne.23377 23749740 PMC4000754

[B10] Pons T. Garraghty P. Ommaya A. Kaas J. Taub E. Mishkin M. (1991). Massive cortical reorganization after sensory deafferentation in adult macaques. Science 252, 1857–1860 10.1126/science.1843843 1843843

[B11] Ramachandran V. S. (1993). Behavioral and magnetoencephalographic correlates of plasticity in the adult human brain. Proc. Natl. Acad. Sci. U.S.A. 90, 10413–10420 10.1073/pnas.90.22.10413 8248123 PMC47787

[B12] Whitt J. L. Petrus E. Lee H.-K. (2014). Experience-dependent homeostatic synaptic plasticity in neocortex. Neuropharmacology 78, 45–54 10.1016/j.neuropharm.2013.02.016 23466332 PMC3796008

